# Therapeutic Salivary Monitoring of Perampanel in Patients with Epilepsy Using a Volumetric Absorptive Microsampling Technique

**DOI:** 10.3390/pharmaceutics15082030

**Published:** 2023-07-27

**Authors:** Michela Palmisani, Elena Tartara, Cecilie Johannessen Landmark, Francesca Crema, Valentina De Giorgis, Costanza Varesio, Cinzia Fattore, Paola Rota, Emilio Russo, Valentina Franco

**Affiliations:** 1 Clinical and Experimental Pharmacology Unit, Department of Internal Medicine and Therapeutics, University of Pavia, 27100 Pavia, Italy; michela.palmisani01@universitadipavia.it (M.P.); francesca.crema@unipv.it (F.C.); 2 IRCCS Mondino Foundation, 27100 Pavia, Italy; cinzia.fattore@mondino.it; 3 Epilepsy Center, ERN Network EpiCare, IRCCS Mondino Foundation, 27100 Pavia, Italy; elena.tartara@mondino.it; 4 Department of Pharmacy, Faculty of Health Sciences, Oslo Metropolitan University, 0316 Oslo, Norway; ceciliel@oslomet.no; 5 The National Center for Epilepsy, Sandvika, ERN Network EpiCare, Oslo University Hospital, 0372 Oslo, Norway; 6 Section for Clinical Pharmacology, Department of Pharmacology, Oslo University Hospital, 0372 Oslo, Norway; 7 Department of Child Neurology and Psychiatry, ERN Network EpiCare, IRCCS Mondino Foundation, 27100 Pavia, Italy; valentina.degiorgis@mondino.it (V.D.G.); costanza.varesio@mondino.it (C.V.); 8 Department of Brain and Behavioral Sciences, University of Pavia, 27100 Pavia, Italy; 9 Department of Biomedical, Surgical and Dental Sciences, University of Milan, 20100 Milan, Italy; paola.rota@unimi.it; 10 Institute for Molecular and Translational Cardiology (IMTC), San Donato Milanese, 20097 Milan, Italy; 11 Science of Health Department, School of Medicine and Surgery, Magna Graecia University of Catanzaro, 88100 Catanzaro, Italy; erusso@unicz.it

**Keywords:** perampanel, LC-MS/MS, therapeutic drug monitoring, saliva, volumetric absorptive microsampling

## Abstract

The objective of this study was to validate a novel assay using the volumetric absorptive microsampling (VAMS) technique combined with liquid chromatography coupled to tandem mass spectrometry (LC-MS/MS) for the determination of the antiseizure medication perampanel in saliva and its clinical applicability in patients with epilepsy. VAMS tips were loaded with 30 μL of saliva and dried for 60 min. Analytes were extracted with methanol. The supernatant was evaporated under a gentle stream of nitrogen and reconstituted with 60 μL of methanol. Separation and quantification were achieved on a monolithic column connected to a mass spectrometer. Calibration curves were linear between 0.5 and 300 ng/mL. Intra- and inter-day accuracy was within 85.6–103.2% and intra-day and inter-day precision did not exceed 12.1%. Perampanel was stable in samples collected by VAMS and stored under different storage conditions. The VAMS-LC-MS/MS method was validated according to internationally accepted criteria and tested in patients with epilepsy who were receiving a combination of perampanel and other antiseizure medications. The method showed adequate bioanalytical performances, holding great potential as an alternative strategy to support domiciliary TDM in patients with epilepsy treated with perampanel according to the simplicity of sample collection.

## 1. Introduction

**Figure 1:**
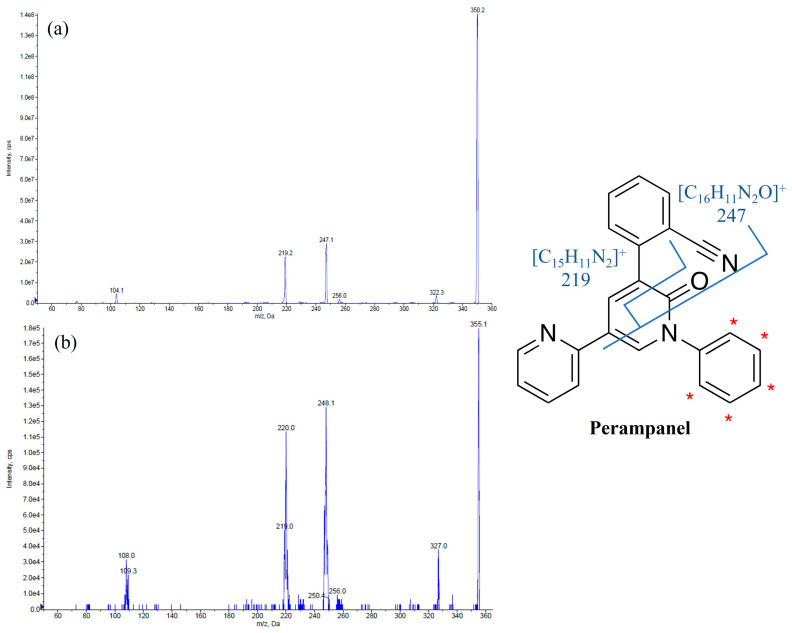
Chemical structures and MS/MS spectra of perampanel (**a**) and the internal standard perampanel-*d*_5_ (**b**). The asterisk (*) was used to indicate [D]-labeling on the internal standard perampanel-*d*_5_ structure. The two primary product ions, *m*/*z* 247 resulting from the formation of the fragment [C_16_H_11_N_2_O]^+^ and *m*/*z* 219 originating from the fragment [C_15_H_11_N_2_]^+^, are also reported [2].

Perampanel (PER) [2-(2-oxo-1-phenyl-5-pyridin-2-yl-1,2-dihdyropyridin-3-yl) benzonitrile hydrate 4:3] is a new antiseizure medication (ASM) acting as selective noncompetitive α-amino-3-hydroxy-5-methyl-4-isoxazole-propionic acid (AMPA) receptor antagonist approved for the treatment of epilepsy (Figure 1) [1].

PER was approved in 2012 by the Food and Drug Administration (FDA) as adjunctive treatment for partial-onset seizures, with or without secondary generalization, in patients aged ≥12 years. PER also received FDA approval in 2015 as adjunctive treatment for primary generalized tonic-clonic seizures in individuals aged 12 years and older diagnosed with genetic (idiopathic) generalized epilepsy. In 2017, the FDA approved PER for use as a standalone treatment for partial-onset seizures with or without secondary generalization in patients aged 12 years and above. In 2018, this indication was extended to encompass pediatric patients aged 4 years and above [3]. In Europe, PER is currently approved for use as adjunctive therapy in patients aged: (i) 4 years and older with partial-onset seizures, with or without secondarily generalized seizures, and (ii) 7 years and older with idiopathic generalized epilepsy who experience primary generalized tonic-clonic seizures [4]. PER is efficiently and quickly absorbed after oral administration, exhibiting a high bioavailability and low systemic clearance [1]. PER binds strongly to proteins, with a binding rate of approximately 95%. In healthy volunteers, the elimination half-life ranged from 53 to 136 h, with an average of 105 h [1]. Patients receiving concomitant enzyme-inducing ASMs exhibited reduced exposure to PER in population studies. In particular, the population pharmacokinetic analysis of patients with partial-onset seizures and primary generalized tonic-clonic seizures revealed that when PER is co-administered with carbamazepine, the total clearance of PER is increased by a factor of three. Similarly, when co-administered with phenytoin or oxcarbazepine, the total clearance of PER is increased by a factor of two. It is important to consider and manage this effect when adding or removing these ASMs from a patient’s treatment schedule [4]. It is crucial to closely monitor the response and drug levels in patients who switch between non-enzyme-inducing and enzyme-inducing ASMs, particularly as the response rates were lower in patients on PER concomitantly treated with CYP3A enzyme-inducing ASMs compared to those taking non-enzyme-inducing ASMs [4]. In fact, the literature findings indicate a correlation between PER plasma levels and seizure control, suggesting that tailoring medication doses through therapeutic drug monitoring (TDM) could be beneficial [5,6].

The putative PER plasma reference range extrapolated from Phase III clinical trials is 180–980 ng/mL [7,8]. In this context, repeated blood sampling can be uncomfortable, especially for children. To alleviate this discomfort and facilitate TDM, utilizing saliva as a non-invasive biological fluid may offer advantages. Salivary samples can serve as an alternative to plasma samples for monitoring purposes since the concentration of many ASMs in saliva mirrors their concentration in plasma [9]. A very recent investigation demonstrated that the PER concentration in saliva was correlated with that in plasma, suggesting the potential application of therapeutic salivary monitoring for PER in patients with epilepsy [10]. In this study, thirty patients receiving PER (2–12 mg/day) were enrolled to assess the usefulness of saliva TDM for PER and the average total levels were 343.02 ng/mL in plasma and 9.74 ng/mL in saliva. Even if TDM is usually performed using plasma samples, unconventional matrixes such as saliva can be easily collected by non-specialized personnel [11]. In this context, the use of new microsampling tools including dried blood spots and volumetric absorptive microsampling (VAMS) have been recently described for some ASMs. Dried blood spot testing is based on the collection of a blood spot onto filter paper to produce a dried sample that can be mailed. This method has been successfully applied for the monitoring of several ASMs [12,13,14,15].

In recent years, VAMS techniques have raised increasing interest as this new approach overcomes the hematocrit bias for blood samples and the issue of inconsistent matrix volume absorption [16,17]. In fact, these handheld devices, which consist of a hydrophilic polymer tip connected to a plastic handler, allow the collection of a fixed and precise volume when in contact with the matrix surface. Compared with blood samples, VAMS technology applied to oral fluids could permit a broader application of TDM by improving sample storage and stability and by facilitating domiciliary self-sampling and shipping procedures. VAMS technology has already been used for the quantitation of cathinone analogues in dried urine, plasma, and oral fluid samples and for the determination of oxycodone and its major metabolites in urine, as well as for some ASMs in blood [17,18,19,20]. More recently, D’Urso and collaborators described a VAMS liquid chromatography–tandem mass spectrometry (LC-MS/MS) method for the analysis of 14 ASMs and two pharmacologically active metabolites in blood taken from patients with epilepsy, with a comparison of these data with the levels determined in plasma [21]. The use of saliva combined with VAMS technology for PER would represent a great advantage to practicing TDM due to the simplicity of the collection method and the use of an alternative and more accessible matrix such as oral fluid.

Based on this background, the purpose of this study was to validate, for the first time, a new LC-MS/MS method to monitor the PER concentrations in the saliva of patients with epilepsy treated with PER using a minimally invasive microsampling system requiring only 30 µL of matrix.

## 2. Materials and Methods

### 2.1. Chemicals, Instrumentation, and LC-MS/MS Parameters

PER was a gift from Eisai (Eisai Co Ltd., Kashima, Japan). Internal standard (IS) PER-*d_5_* and Mitra^®^ VAMS devices were purchased from B.S.N. (B.S.N. srl R&D Laboratory, Castelleone, Italy). Ultrapure water was obtained by means of a Millipore-Q-plus system (Millipore, Milan, Italy). LC-MS grade methanol, acetonitrile, and 99% formic acid were purchased from VWR (VWR International, Radnor, PA, USA). Dimethylsulfoxide (≥99.5%) was purchased from Sigma Aldrich (Sigma Aldrich, St. Louis, MO, USA). For the validation of the method, drug-free human saliva was obtained from healthy volunteers. Participants were instructed to abstain from consuming food or beverages for a duration of 30 min prior to sample collection and to rinse their mouths with plain water. Saliva was obtained using an unstimulated passive drool technique for sampling. The oral fluid was then aspirated with a syringe and transferred into 2 mL polypropylene tubes.

The LC-MS/MS system used for the analyses was composed of a Sciex ExionLC 100 integrated system (Applied Biosystems Sciex, Darmstadt, Germany) coupled to a SCIEX API 3200 QTRAP^®^ triple quadrupole mass spectrometer (Applied Biosystems Sciex, Darmstadt, Germany) equipped with an electrospray ionization source operating in positive ion mode. A C18 column (Onyx, 100 × 3 mm i.d., Phenomenex, Torrance, CA, USA) heated at 25 °C was chosen to perform the analyses in order to obtain the best chromatographic condition. The mobile phase A (0.1% acid formic in water) and the mobile phase B (methanol) flowing at 0.9 mL/min were used with the following gradient elution program: A:B = 98:2 (*v*/*v*) from 0 to 2 min, A:B = 25:75 (*v*/*v*) from 2.01 to 5 min, and A:B = 98:2 (*v*/*v*) from 5.01 to 7 min. The mass spectrometry acquisition was performed in multiple reaction monitoring modes. The optimized parameters of the instrument included ion spray voltage, curtain gas, ion source gas 1, ion source gas 2, and temperature with the following setup: 5500 V, 35 psi, 45 psi, 50 psi, and 600 °C, respectively. Nitrogen flow was generated by a gas generation system (nitrogen generator model Genius ABN2ZA, PEAK Scientific., Scotland). LC-MS/MS control and data acquisition were performed using Analyst software version 1.6.3, whereas LC-MS/MS data processing was carried out using MultiQuant software version 3.0.2 (Applied Biosystems Sciex, Darmstadt, Germany). Table 1 reports the MS/MS optimized parameters for PER and IS.

### 2.2. Preparation of Stock and Working Solutions

Stock solutions were prepared in acetonitrile at the following concentration: PER 1 mg/mL and IS 1 µg/mL and stored at −20 °C. Dilutions of the PER stock solution were made in dimethylsulfoxide (≥99.5%) to prepare working solutions with concentrations of 5, 50, 200, 500, 1000, 2000, and 3000 ng/mL for calibrators and 40, 800, and 2500 ng/mL for quality control (QC) samples. The working calibrators and quality control solutions were stored at a temperature of 4 °C for a maximum period of 21 days. The working IS solution (140 ng/mL) was prepared in methanol from the stock solution and stored at −20 °C. For calibrators and QC samples, aliquots of 5 μL of working solutions were added to 45 μL of blank saliva.

### 2.3. Sample Preparation

VAMS samples were prepared by touching the fluid surface with the tip of the device to absorb 30 μL of the sample. Subsequently, the loaded VAMS device was dried for 60 min at room temperature and placed into a plastic 1.5 mL tube. A volume of 30 µL of the IS and 470 µL of methanol were added. The sample was then vortexed for 10 s and shaken for 10 min. Samples were additionally incubated for 10 min at 50 °C and sonicated for 10 min. The VAMS tip was removed from the plastic tube and the sample was centrifuged at 17,000× *g* at 4 °C for 10 min using a Micro Star 17R centrifuge (VWR International, Radnor, PA, USA). A 440 µL aliquot of the supernatant was evaporated at room temperature under a gentle stream of nitrogen and reconstituted with 60 µL of methanol. Finally, the reconstituted sample was transferred into the autosampler maintained at room temperature and a volume of 20 µL was injected into the LC-MS/MS system.

### 2.4. Method Validation

The validation of the method was performed according to the European Medicines Agency recommendations on bioanalytical method validation [22].

For linearity, calibration curves were prepared using calibrators containing PER at 0.5, 5, 20, 50, 100, 200, and 300 ng/mL and were constructed by plotting the PER/IS peak area ratios against the nominal PER concentrations in the calibrators. The calibration curves were computed without weighing and all data underwent linear regression analysis. The correlation coefficient was employed as an indicator of the quality of the fit. To evaluate the sensitivity of the method, the limit of quantitation (LOQ) and the limit of detection (LOD) were determined. The LOQ was defined as the minimum concentration of calibrators that yielded a signal-to-noise ratio of at least 10. It was considered acceptable if the coefficient of variation (CV%) was below 20% and the accuracy fell within ±20%. On the other hand, the LOD was determined as the concentration of calibrators with a signal-to-noise ratio of at least three. Within-run and between-run precision and accuracy were evaluated at four concentrations corresponding to the LOQ, low QC, medium QC, and high QC, and were determined by measuring the LOQ and QC samples on one day (*n* = 5) and the LOQ and QC samples on three different days (*n* = 15), respectively. Precision was expressed as CV% and the assay was considered acceptable if the CV at each concentration was less than 15%. The accuracy was determined by comparing the average values of the LOQ and QCs assay results with the nominal concentrations and results should be within 15% of the nominal values. The evaluation of extraction recovery was conducted using four different concentrations (LOQ, low QC, medium QC, and high QC) and it was assessed by comparing the peak area of PER obtained in VAMS spiked samples before extraction (*n* = 5) and the peak area of PER obtained in VAMS spiked samples after extraction (*n* = 5). 

In addition, the matrix effect was evaluated by determining the matrix factor (MF) for each analyte and IS in every batch of the matrix. This involved calculating the ratio of the peak area obtained when the analyte was present in the matrix (measured by analyzing a blank matrix spiked with the analyte after extraction) to the peak area obtained when the analyte was in a pure solution without the matrix. Additionally, the IS-normalized MF was calculated by dividing the MF of the analyte by the MF of the IS. To evaluate the consistency of the MF, the CV for the IS-normalized MF was calculated using data from six different matrix lots. It was required that the CV did not exceed 15% as a criterion for acceptability. This assessment was performed at both low and high QC levels. To assess the stability of PER in VAMS and working solutions, the low and high QC samples (*n* = 4) were used. Assay values obtained from fresh extracts were compared with values obtained from extracts stored at the same concentration level under different conditions: 72 h at room temperature, −20 °C for one month, and after three freeze–thaw cycles. Furthermore, the stability of the extracts was evaluated by leaving the extracted samples in the autosampler for 24 h at room temperature. The stability of PER in working solutions was examined by storing them at 4 °C for 21 days and comparing their response against freshly prepared working solutions. The selectivity of the method was evaluated by examining the absence of interfering peaks at the retention time of PER and the IS in human saliva samples collected from six different healthy individuals. Carry-over was determined by analyzing six consecutive runs of extracted blank samples following the highest calibrator. It was considered acceptable if the peak areas of PER and the IS were not greater than 20% of the LOQ and 5% for the IS.

### 2.5. Clinical Application

The method’s suitability for TDM was demonstrated through the analysis of human saliva VAMS samples collected from patients with epilepsy who were undergoing treatment with PER in combination with other ASMs. Participants were recruited from the Child Neurology and Psychiatry Unit and the Epilepsy Center of the IRCCS Mondino Foundation in Pavia, Italy. The local Ethics Committee granted approval for the study (Reference N°: P-20170012031). Written informed consent was obtained from all subjects or parents of subjects participating in the study. The samples were obtained during the steady state period, approximately 12 h after the evening dose, and stored at −20 °C until the time of analysis.

### 2.6. Data Analysis

The concentrations of analytes were quantified and presented as ng/mL. Values were reported as the means ± SD. The stability parameters were compared using repeated measures analysis of variance (ANOVA). A *p*-value of ≤ 0.05 was considered indicative of statistical significance. All statistical analyses were conducted using GraphPad Prism version 8.2.1 (GraphPad Software Inc., San Diego, CA, USA).

## 3. Results

### 3.1. Chromatographic Separation

**Figure 2:**
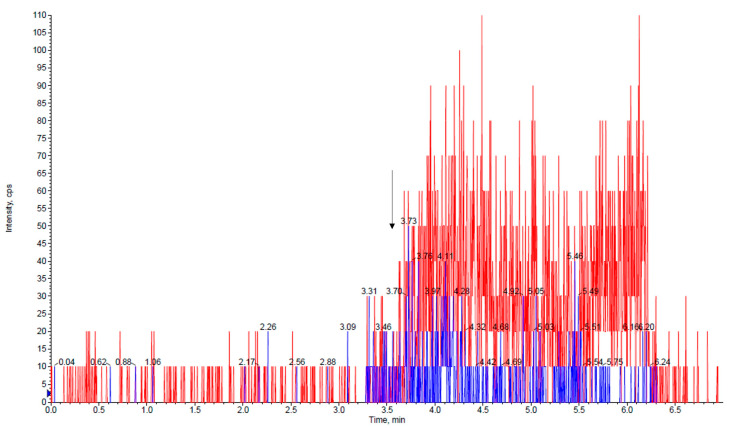
Chromatogram of a VAMS blank saliva sample. The arrow indicates the retention time of the perampanel and the IS. PER, perampanel (blue line); PER-*d*_5_, perampanel-*d*_5_ (red line).

**Figure 3:**
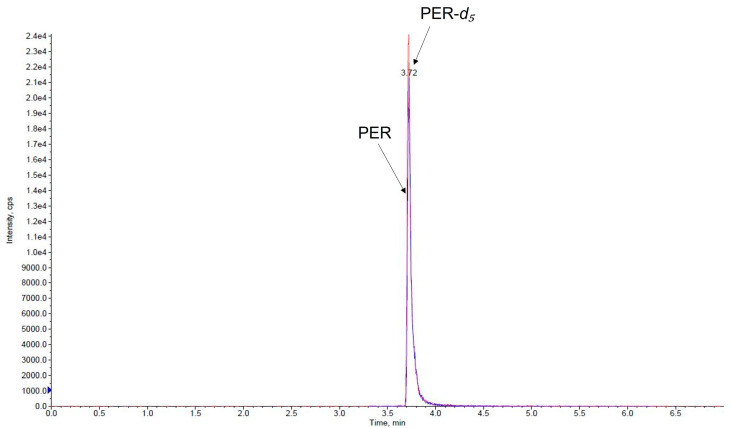
Chromatogram of a VAMS saliva sample spiked with a medium QC (80 ng/mL). PER, perampanel (blue line); PER-*d*_5_, perampanel-*d*_5_ (red line).

**Figure 4:**
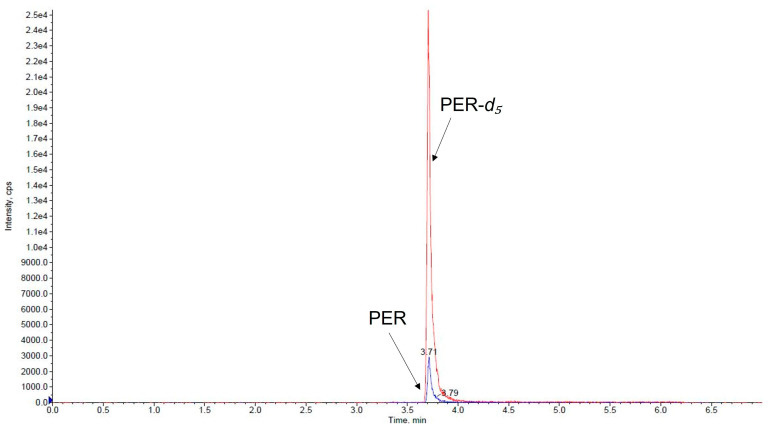
Chromatogram of a VAMS saliva sample from a patient who was taking perampanel at a dosage of 6 mg/day. The salivary concentration of perampanel measured was 6.30 ng/mL. PER, perampanel (blue line); PER-*d*_5_, perampanel-*d*_5_ (red line).

Under the applied chromatographic condition, the retention time of PER and IS was 3.7 min. Representative chromatograms of a VAMS blank saliva sample, a medium QC VAMS saliva sample, and a VAMS saliva sample taken from a subject treated with PER are depicted in Figure 2, Figure 3 and Figure 4.

### 3.2. Method Validation

**Figure 5:**
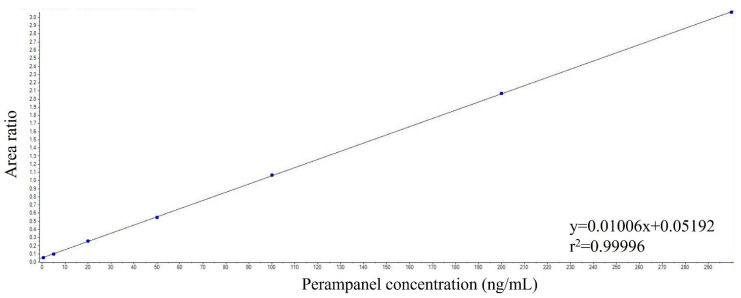
Representative calibration curve for perampanel measured in VAMS samples.

The calibration curves displayed linearity within the concentration range of 0.5–300 ng/mL, exhibiting a coefficient of correlation equal to or greater than 0.9999 (Figure 5). The calibration curves were determined on five different days, and the calculated average slope across all five curves was 0.011. Furthermore, any potential cross-signal interference caused by chemical impurities in the reference standard, isotopic interference, or crosstalk within the mass spectrometer between PER and the IS was excluded.

The mean ± SD values for the within-run and between-run precision and accuracy are presented in Table 2. The intra-day and inter-day precision did not exceed 12.1% and the intra-day and inter-day accuracy was within 85.6–103.2%. The LOQ and LOD were established at 0.5 ng/mL and 0.05 ng/mL, respectively. In particular, the intra-day precision and accuracy values of the LOQ were 7.6% and 85.6%, whereas the inter-day precision and accuracy values were 12.1% and 93.3%, respectively (Table 2). The back-calculated concentrations of the calibration standards were within a range of ±15% of the expected values. Additionally, a minimum of 75% of the calibration standards met these criteria. For the QC samples, at least 67% of them met the ±15% deviation from the expected values. Overall, the obtained values were in line with the EMA guidelines on bioanalytical method validation [22]. The mean extraction recoveries for PER at the LOQ, low QC, medium QC, and high QC levels were 97.2%, 81.3%, 98.7%, and 96.5%, respectively. The mean IS-normalized MF values obtained for low and high QC were 0.87 ± 0.03 and 0.94 ± 0.03, respectively. The matrix effect was considered negligible since the obtained CV values were below 3.9%.

PER stability was evaluated under different storage conditions as shown in Table 3. Specifically, stability was proven for VAMS saliva samples stored at room temperature for 72 h, at −20 °C for 1 month, and after three freeze–thaw cycles. Confirmation of autosampler stability was obtained for samples that were kept at room temperature for 24 h. The stability of PER in working solutions was confirmed when stored at 4 °C for 21 days, with a percentage of variation of the mean peak area ratio ranging from −9.9 to 3.5.

No interfering peaks were observed in the saliva VAMS samples collected from six different healthy individuals around the retention time of PER and IS. Similarly, the saliva VAMS samples obtained from patients receiving concomitant ASMs (cannabidiol, carbamazepine, ethosuximide, lamotrigine, phenobarbital, topiramate, and vigabatrin) also did not exhibit any interfering peaks. Carry-over was negligible for both PER and the IS as peak areas of analytes were not greater than 20% of the LOQ and 5% of the IS.

### 3.3. Clinical Application

The method described above was applied for the determination of PER concentrations in samples collected by VAMS from one patient treated with three different dose levels, namely 4, 6, and 8 mg/day, one patient treated with the same stable dose (4 mg/day), and two additional patients on PER treatment as shown in Table 4. As per guidelines, the analysis included the evaluation of a blank sample, calibration standards at seven different concentrations, as well as a duplicate analysis of low, medium, and high QC samples. Accuracy values were 102.1%, 108.7%, and 107.4% for low, medium, and high QC samples, respectively. Precision values were 9.5%, 5.4%, and 5.1% for low, medium, and high QC samples, respectively. The PER concentration in the analyzed saliva VAMS samples ranged from 1.25 to 9.95 ng/mL. Considering the obtained preliminary data, we did some additional testing by adding a QC sample at 1 ng/mL. The intra-day (*n* = 5) precision and accuracy values were 2% and 95.8%, respectively, while the inter-day (*n* = 10) precision and accuracy values were 5.7% and 99.1%, respectively.

## 4. Discussion

Due to significant variability in the pharmacokinetics of different ASMs, TDM plays a crucial role in determining the appropriate dosage for epilepsy treatment. In modern epilepsy treatment, TDM is increasingly used to identify the optimal blood concentration for an effective therapeutic response in each individual patient. Subsequent measurements are then taken to ensure that this concentration is maintained over time, thereby preventing potential declines in clinical response due to variations in compliance or drug absorption and elimination processes.

To ensure clinical utility and efficacy in clinical practice, drug concentration measurements for TDM should ideally occur under steady-state conditions, reflecting stable therapy. However, this necessitates venous sampling, considered the reference standard, to be conducted in a hospital setting, typically in the morning prior to the treatment administration. Performing TDM sampling demands specialized hospital staff and can be challenging to perform in pediatric patients or uncooperative patients who have to face long journeys to reach specialized tertiary care centers. Recently, research has focused on minimizing the blood sample volume by implementing microsampling techniques for drug bioanalysis. This innovative approach offers several advantages, including improved patient comfort, simplified sample collection, and the possibility of remote collection.

In the present original protocol, a novel analytical method has been validated according to EMA criteria [22] for the salivary monitoring of PER using innovative VAMS technology coupled with LC-MS/MS analysis. The developed approach was tested for the first time in the saliva (30 µL) of patients with epilepsy and found to be reliable for the quantification of PER in the oral fluid collected by VAMS. The results elucidate the potential of this approach as an alternative strategy to support TDM in patients with epilepsy. The findings from stability studies provide evidence in favor of employing VAMS technology for the purpose of sample storage and transportation, highlighting their advantageous features. Treatment with PER may thus be easily followed according to the simplicity of sample collection through saliva monitoring in a small sample volume. This may serve as an example for various other drugs in future studies. The utilization of readily available non-invasive biological fluid, such as saliva collected using innovative microsampling technologies, can provide significant advantages in terms of therapeutic monitoring. It is well established that the concentration of several ASMs in saliva mirrors their concentration in plasma/serum. This equivalence enables the substitution of saliva samples for plasma/serum samples when monitoring drug levels [11]. The advantages of the use of saliva are as follows: (i) for many ASMs, the saliva concentration reflects the free, pharmacologically active concentration in serum; (ii) samples may be drawn repetitively; (iii) it is a non-invasive approach that does not require specialized expertise for performing the collection. Furthermore, it is preferred by many patients, including children and their parents as well as the elderly [11].

The main novelty of the present original research is the use of VAMS loaded with saliva with significant advantages for the simplicity of the collection method and the use of an alternative and more accessible matrix such as oral fluid. The selection of oral fluid as an alternative matrix and the sample collection by VAMS was based on the following considerations: (i) there is a significant correlation between the PER concentrations in saliva and plasma, indicating that saliva samples can be utilized for TDM purposes; (ii) salivary determination could be particularly suitable for children treated with PER, in order to avoid potential discomfort due to recurrent venipunctures and to monitor the occurrence of side effects during the titration period; (iii) VAMS technology can be performed at home and dried samples can be sent to the laboratory by mail.

PER is a drug that is increasingly used in refractory epilepsy, and its efficacy and tolerability were recently reviewed in a large, pooled analysis of 44 studies [23]. Results from almost 5000 patients showed a mean retention time of 11 months, a 50% responder rate of 60% at 12 months, and 50% of patients reported adverse effects [23].

The developed assay was successfully used to test salivary samples collected by VAMS from patients with epilepsy treated with PER and obtained solid and satisfactory results. However, more studies with a large number of patients are required to assess the correlation between saliva PER levels and saliva PER level in samples collected by VAMS and the PER therapeutic range in saliva. The evaluation of serum concentrations and efficacy has been shown to be variable [24]. In a recent study, the possible relationship between serum concentrations, pharmacokinetic variability, and evaluation of efficacy and tolerability was studied in patients with refractory epilepsy. However, it was difficult to draw conclusions on the correlations between serum levels and efficacy and tolerability due to low sample size and the large variability between patients [25]. Further studies are also required to evaluate the relationship between salivary PER concentrations, antiseizure efficacy, and adverse effects.

In our study, the combination of VAMS and saliva sampling provided a simple methodology for implementation in clinical practice. As previously experienced, the sampling of large volumes of saliva was a practical challenge [26], whereas the small volume needed here is easy to collect. The small number of samples from patients included in this study gives an indication of use in larger settings. In fact, VAMS technology offers an intriguing feature which is the ability for individuals to perform self-sampling at home. By utilizing VAMS, patients can safely take their own saliva samples and send them to the laboratory via regular mail. This study may therefore serve as an example for further investigations with other ASMs.

## 5. Conclusions

The innovative VAMS technology presented in this paper was demonstrated to be precise and accurate and to provide optimal results from only 30 µL of oral fluid, suggesting the potential for future TDM implementation through domiciliary sampling procedures. In this context, VAMS technology could pave the road in the near future to a patient-centered approach transition where patients are able to collect a fixed volume of saliva at their homes without the need for hospital visits.

## Figures and Tables

**Table 1:** Optimized MS/MS parameters for PER and IS.

Analyte	RT (min)	Q1 Mass (*m*/*z*)	Q3 Mass (*m*/*z*)	DP (V)	CE (V)	CXP (V)
Perampanel	3.7	350.1	247.1	52	47	10
Perampanel-*d*_5_	3.7	355.1	248.1	52	45	10

Abbreviations: collision cell exit potential, CXP; collision energy, CE; declustering potential, DP; retention time, RT.

**Table 2:** Intra-day and inter-day precision (CV%) and accuracy (%) of PER measured in saliva samples collected by VAMS and spiked with the LOQ, low QC, medium QC, and high QC working solutions.

Parameter	LOQ 0.5 ng/mL	Low QC 4 ng/mL	Medium QC 80 ng/mL	High QC 250 ng/mL
		Intra-day precision and accuracy (*n* = 5)		
Measured concentration (ng/mL)	0.4 ± 0.03	3.6 ± 0.2	81.3 ± 2.3	254.6 ± 5.0
Precision (CV%)	7.6	6.6	2.9	2.0
Accuracy (%)	85.6	90.6	101.6	101.9
		Inter-day precision and accuracy (*n* = 15)		
Measured concentration (ng/mL)	0.5 ± 0.1	4.0 ± 0.5	81.3 ± 3.0	257.9 ± 15.6
Precision (CV%)	12.1	11.8	3.7	6.0
Accuracy (%)	93.3	100.2	101.6	103.2

**Table 3:** PER stability in VAMS saliva samples spiked with low and high QC working solutions.

Stability Condition	Low QC 4 ng/mL	High QC 250 ng/mL
Fresh samples (ng/mL)	3.7 ± 0.3	256.1 ± 4.5
Precision (CV%)	6.9	1.8
Accuracy (%)	91.7	102.4
72 h at room temperature (ng/mL)	3.9 ± 0.2	253.9 ± 6.3
Precision (CV%)	5.7	2.5
Accuracy (%)	96.3	101.6
1 month at −20 °C (ng/mL)	3.7 ± 0.2	265.0 ± 4.6
Precision (CV%)	5.7	1.7
Accuracy (%)	92.3	106.0
Three freeze–thaw cycles (ng/mL)	3.9 ± 0.1	257.3 ± 9.9
Precision (CV%)	1.4	3.9
Accuracy (%)	98.4	102.9
24 h at room temperature in autosampler	3.7 ± 0.2	256.0 ± 7.1
Precision (CV%)	5.1	2.8
Accuracy (%)	92.3	102.4

**Table 4:** Characteristics of the four patients included their salivary PER concentrations in the analyzed samples collected by VAMS.

Patient Number	Sex	Dose of PER (mg/day) *	Concomitant ASMs	Saliva PER Concentration (ng/mL)
1	F	4	CBZ, PB, TPM	1.55
1	F	4	CBZ, PB, TPM	1.25
2	F	4	PB, LTG	2.75
2	F	6	PB, LTG, TPM, CBD	6.30
2	F	8	PB, LTG, TPM, CBD	9.95
3	F	4	VGB, CBZ, PB	1.88
4	M	6	ESM	4.39

Abbreviations: F, female; M, male; ASMs, antiseizure medications; CBD, cannabidiol; CBZ, carbamazepine; ESM, ethosuximide; LTG, lamotrigine; PER, perampanel; PB, phenobarbital; TPM, topiramate; VGB, vigabatrin. * The maintenance dose taken at the time of sample collection.

## Data Availability

The raw data that support the findings of this article are available on the ZENODO repository https://doi.org/10.5281/zenodo.8123344.
